# How Childhood Harshness Affects Adolescent Mental Health: The Role of Basic Psychological Needs

**DOI:** 10.3390/bs16030338

**Published:** 2026-02-28

**Authors:** Ru Zhou, Jiaxu Zhao, Xiaosong Gai, Xiaoming Liu, Kai Gao

**Affiliations:** 1 Faculty of Education, Northeast Normal University, Changchun 130024, China; zhour@nenu.edu.cn; 2 Psychological Research and Training Center, Dalian Education University, Dalian 116021, China; 3 School of Psychology, Northeast Normal University, Changchun 130024, China; zhaojiaxu@nenu.edu.cn (J.Z.); gaixs669@nenu.edu.cn (X.G.); liuxm@nenu.edu.cn (X.L.); 4 Research Center of Mental Health Education in Northeast Normal University, Key Research Institute of Humanities and Social Science in Universities in Jilin Province, Changchun 130024, China; 5 Institute of Educational Sciences, Dalian Education University, Dalian 116021, China

**Keywords:** harshness, psychological needs, mental health, adolescence

## Abstract

Accumulating evidence suggests that childhood adversity can have long-lasting effects on adolescent mental health. However, less is known about the specific impact of childhood harshness on mental well-being. This study builds on Life History Theory and Basic Psychological Need Theory to explore the relationship between childhood harshness and adolescent mental health. Study 1, utilizing cross-sectional data from 1491 adolescents (age: M = 13.74, SD = 0.73), found that childhood harshness negatively predicted mental health, with basic psychological need satisfaction serving as a significant mediator. Study 2 further examined the temporal effects in a new sample of 918 adolescents (age: M = 12.62, SD = 0.61) using a three-wave longitudinal design, providing stronger support for the temporal ordering of these associations. These results underscore both the immediate and long-term effects of childhood harshness on mental health and suggest that interventions targeting basic psychological need satisfaction may help mitigate these negative impacts.

## 1. Introduction

Adolescence is a critical developmental period marked by significant psychological and emotional changes. However, recent years have seen adolescents grappling with unprecedented challenges that exacerbate mental health issues, making them more complex than the typical stressors of this age group (Conte et al., 2025). For instance, the suicide rate among children aged 5–14 has risen by an average of 10% annually (Zhao et al., 2023). Since the early 2010s, accumulating data from various countries have pointed to a worsening trend in adolescent mental health, often described as a “mental illness epidemic” (K. M. Keyes & Platt, 2024; Twenge, 2020). These alarming trends highlight the urgent need to identify and understand the underlying psychological mechanisms that contribute to adolescent mental health challenges, particularly those related to early life experiences such as childhood harshness.

Early life experiences can have a “long-arm effect”, where negative childhood experiences continue to influence social adaptation and mental health long into adulthood (Kang et al., 2021; Stoltenborgh et al., 2015). Research has shown that childhood harshness is one of the critical factors influencing adolescent mental health development (Haimov et al., 2022). However, existing research has largely focused on general adversity indices and negative psychopathological outcomes, with relatively limited attention to the mechanisms through which ecological harshness influences broader mental health functioning, including positive dimensions (Carvalho et al., 2020). As Peterson (Park & Peterson, 2009) suggested, “what is good in life is not simply the absence of what is problematic.” Therefore, exploring the mechanisms linking childhood harshness to mental health is of considerable theoretical and practical importance. This study aims to address this gap by examining how childhood harshness impacts adolescent mental health, with a focus on the mediating role of basic psychological need satisfaction. By utilizing both cross-sectional and longitudinal approaches, this research seeks to clarify how unmet psychological needs during childhood may contribute to long-term mental health outcomes. The findings from this model may offer a positive perspective for psychological interventions for adolescents who have experienced childhood harshness.

### 1.1. Childhood Harshness and Adolescents’ Mental Health

Harshness, as conceptualized within the evolutionary developmental framework, refers specifically to environmental conditions characterized by elevated morbidity and mortality risk and chronic scarcity of material resources (Ellis et al., 2009; Lin et al., 2024). It reflects the degree to which the ecological context signals shortened life expectancy and limited energetic availability (Usacheva et al., 2022). Importantly, harshness is conceptually distinct from unpredictability, which captures stochastic and erratic changes in environmental conditions over time, and from maltreatment, which refers to specific harmful interpersonal experiences such as abuse or neglect (McLaughlin et al., 2021). Although these constructs often co-occur under the broader umbrella of childhood adversity, they represent theoretically separable dimensions of early experience.

According to Life History Theory, individuals adaptively calibrate their developmental strategies in response to ecological cues regarding resource availability and survival risk (A. Yang et al., 2022). When early environments are characterized by high harshness, the developmental system may shift toward strategies prioritizing immediate resource acquisition and short-term survival over long-term investment in growth and social bonds (Szepsenwol et al., 2022). In contrast, relatively resource-abundant and low-mortality contexts are more likely to promote slower life history strategies that emphasize future-oriented planning and stable interpersonal relationships (Griskevicius et al., 2011). Importantly, the psychological consequences of harshness arise not merely from discrete negative events, but from chronic exposure to ecological signals of scarcity and survival threat embedded in the broader context (Ellis et al., 2022). Even in the absence of acute adverse incidents, such environments provide limited opportunities for sustained emotional security and long-term relational investment (Szepsenwol et al., 2019). As a result, although these adaptive strategies may serve short-term survival functions, they are unlikely to foster positive developmental outcomes in the long run. Previous research has shown that prolonged exposure to harsh ecological conditions is associated with poorer emotional regulation capacities and impaired interpersonal functioning, which in turn increase adolescents’ vulnerability to mental health difficulties (Dickerson & Quas, 2021; Sroufe, 2005). Based on this theoretical framework, we hypothesize that higher levels of childhood harshness will be associated with poorer adolescent mental health.

### 1.2. Basic Psychological Need Satisfaction as a Mediator

According to Basic Psychological Need Satisfaction (BPNS) Theory, individuals have three fundamental psychological needs: autonomy, competence, and relatedness, which are essential for optimal psychological functioning and well-being (Martela & Ryan, 2024; Ryan & Deci, 2000). Autonomy refers to the ability to make one’s own decisions without external pressure, competence involves feeling effective in interacting with the environment, and relatedness is the sense of being cared for and loved by others. The satisfaction of these needs is critical for healthy development (Van den Broeck et al., 2016). BPNS is strongly influenced by the external environment (Deci & Ryan, 2000). A supportive and nurturing environment can promote the fulfillment of these needs, while a harsh environment hinder it (Daelmans et al., 2021).

In the context of childhood adversity, unmet basic psychological needs may mediate the relationship between childhood harshness and adolescent mental health. On one hand, childhood harshness can disrupt the satisfaction of autonomy, competence, and relatedness. A meta-analysis revealed that adverse childhood experiences were strongly associated with lower emotional well-being (Yeo et al., 2024), and one study also showed that childhood harshness significantly and negatively predicted the overall level of BPNS in Chinese adolescents (Geng et al., 2022). On the other hand, the satisfaction of basic psychological needs may lead to better mental health outcomes, such as reduced depression and anxiety (Ryan et al., 2008). A high-risk environment that fails to meet these needs may exacerbate behavioral problems and internalizing issues in adolescents (Li et al., 2016). A meta-analysis further supports this, showing that the satisfaction of basic psychological needs is linked to more positive affect, less negative affect, and fewer symptoms of depression and anxiety in health-related contexts (Ng et al., 2012). Therefore, BPNS plays a crucial mediating role in the relationship between childhood harshness and adolescent mental health, as unmet psychological needs may serve as a key mechanism through which childhood harshness impacts mental well-being.

### 1.3. The Present Study

This study employed a retrospective approach to examine the association between childhood harshness and mental health among Chinese junior high school students, and to investigate the mediating role of basic psychological need satisfaction. Grounded in life history theory, we posited that more severe experiences of childhood harshness would be associated with poorer mental health outcomes in adolescence. In Study 1, we collected cross-sectional data from 1491 junior high school students to examine the relationship between childhood harshness and mental health, as well as the potential mediating role of basic psychological need satisfaction. However, due to the cross-sectional design, temporal inferences could not be established. Drawing on basic psychological needs theory, we further proposed that basic psychological need satisfaction represents a key psychological mechanism linking childhood harshness to adolescent mental health. To examine temporal dynamics more rigorously, Study 2 employed a three-wave longitudinal design with an independent adolescent sample to test whether childhood harshness predicts later mental health through subsequent basic psychological need satisfaction.

Accordingly, the present research tested the following hypotheses:

**Hypothesis** **1.** *Childhood harshness will negatively predict mental health.*

**Hypothesis** **2.** *Basic psychological need satisfaction will mediate the relationship between childhood harshness and mental health.*

**Hypothesis** **3.** *Childhood harshness at Time 1 will indirectly predict mental health at Time 3 through basic psychological need satisfaction at Time 2, demonstrating a temporal mediating effect.*

## 2. Study 1

The aim of Study 1 was to evaluate the potential predictive role of childhood harshness on adolescents’ mental health, as well as the mediating role of basic psychological needs. To achieve this, we adopted a cross-sectional design. Given the existing literature suggesting that these demographic factors may exert an impact on individuals’ mental health (Matud et al., 2019; Springer et al., 2011), we considered gender and age as control variables.

### 2.1. Method

#### 2.1.1. Participants and Procedures

We used convenience sampling to recruit participants from a public middle school located in an urban area of Dalian, Liaoning Province, China. The study was approved by the Ethics Committee of the School of Psychology at the local university (approval number: 2024014). Written informed consent was obtained from the students, their legal guardians, and the school prior to data collection. Data were collected in November 2024 from Grade 8 and Grade 9 students (equivalent to second and third year of junior high school in China). A total of 1491 middle school students participated in Study 1, including 49.03% females, with ages ranging from 12 to 16 years (M = 13.74, SD = 0.73). Among them, 1487 students resided in urban areas. The sample comprised 772 Grade 8 students and 719 Grade 9 students. The survey was administered online via the platform (www.wjx.cn). Students completed the questionnaires in their classrooms and were informed that participation was voluntary and that they could withdraw at any time. The survey included measures of childhood harshness, basic psychological need satisfaction, mental health, and demographic information. Experienced teachers supervised the data collection process and checked the completeness of the questionnaires after completion.

#### 2.1.2. Measures

##### Childhood Harshness

The Childhood Harshness Scale developed by Maranges et al. (2022) was used, with the Chinese version translated and revised by Lin et al. (2024). The scale consists of 11 items, rated on a 7-point Likert scale, with scores ranging from 1 (strongly disagree) to 7 (strongly agree). An example item from the scale is: “Although my parents work very hard, my family rarely has enough money to buy luxury goods.” The scores for these items are summed and averaged, with higher scores indicating that an individual experienced a harsher early childhood environment with fewer available resources and lower economic status (Maranges et al., 2022). This scale has been administered to adolescents and has demonstrated good reliability and validity (Song et al., 2024; Zeigler-Hill et al., 2024). In this study, the Cronbach’s alpha for this scale was measured with a coefficient of 0.87.

##### Basic Psychological Need Satisfaction

The Basic Psychological Need Satisfaction in Relationships Scale (La Guardia et al., 2000), adapted into Chinese by Du et al. (2020), was used to assess the degree of basic need satisfaction in specific relationships. The scale evaluates an individual’s experience of basic need satisfaction in relationships with their spouse, best friend, or parents. In this study, the scale was used to assess adolescents’ basic psychological need satisfaction in the parent–child relationship, with the phrase “When I am with XXX” replaced by “When I am with my parents” (La Guardia et al., 2000). The scale consists of three dimensions: autonomy, competence, and relatedness, each containing 3 items, for a total of 9 items. A representative item is: “When I am with my parents, I can freely be myself.” The scale uses a 7-point Likert scale (1 = “Not at all true,” 7 = “Completely true”). Higher scores indicate a higher level of satisfaction of the adolescent’s basic psychological needs. In the present study, the Cronbach’s alpha was 0.89 for the overall scale, with subscale reliabilities of ω = 0.75 for autonomy, 0.69 for competence, and 0.75 for relatedness.

##### Mental Health

Mental health was assessed using the Chinese version of the Mental Health Continuum–Short Form (MHC-SF; C. L. Keyes, 2007), adapted by Guo et al. (2015). The MHC-SF is a shortened version of the original Mental Health Continuum–Long Form (MHC-LF). The 14 items measure three components: emotional well-being (3 items), psychological well-being (6 items), and social well-being (5 items). Responses are rated on a 6-point Likert scale, from “never” (0) to “every day” (5). The total score, ranging from 0 to 70, reflects overall well-being, with higher scores indicating better mental health. The MHC-SF has been employed with adolescents, demonstrating good reliability and validity in previous studies (Khazaei et al., 2023). In the present study, the Cronbach’s alpha for the overall scale was 0.95. The reliabilities of the emotional, social, and psychological well-being subscales were ω = 0.92, 0.86, and 0.92, respectively.

#### 2.1.3. Data Analysis

Data analyses were conducted using SPSS 26.0 and Mplus 8.3. Descriptive statistics and Pearson correlation analyses were performed in SPSS 26.0 to examine the relationships among childhood harshness, basic psychological need satisfaction, and mental health. Independent-samples *t* tests were used to assess gender differences. Multiple regression analyses were then conducted to examine the predictive effects of childhood harshness and basic psychological need satisfaction on mental health while controlling for demographic variables (gender and age). Finally, structural equation modeling was performed in Mplus 8.3 to test the mediating role of basic psychological need satisfaction in the association between childhood harshness and mental health, with indirect effects evaluated using a bias-corrected bootstrap method.

### 2.2. Results

#### 2.2.1. Descriptive Statistics

Table 1 presents the correlations among all assessed variables. Childhood harshness was significantly and negatively correlated with the three dimensions of basic psychological need satisfaction as well as with the three dimensions of mental health (all *p* < 0.001). In contrast, all dimensions of basic psychological need satisfaction were significantly and positively correlated with the dimensions of mental health (all *p* < 0.001).

Independent-samples *t* tests were conducted to examine gender differences across the study variables (see Table 2). The results indicated significant gender differences in childhood harshness, relatedness, and emotional well-being, with males reporting higher levels than females.

#### 2.2.2. Regression Analysis

Multiple linear regression analyses (enter method) were performed, with mental health (emotional, social, and psychological well-being) as the dependent variables and childhood harshness together with the dimensions of basic psychological need satisfaction (autonomy, competence, and relatedness) as independent variables. As shown in Table 3, the findings indicate that childhood harshness negatively predicted all dimensions of mental health. In contrast, autonomy, competence, and relatedness satisfaction positively predicted mental health outcomes, with competence satisfaction exhibiting the strongest unique predictive effect across the three models. The partial r values reflect the unique strength of association between each predictor and mental health after controlling for the other variables in the regression models.

#### 2.2.3. Mediation Analysis

**Figure 1:**
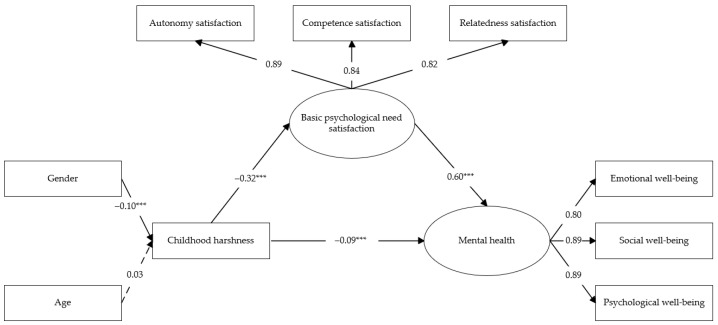
A mediation model of childhood harshness, basic psychological need satisfaction, and mental health in Study 1. Note. *** *p* < 0.001.

Building on the above analyses, we further examined the mediating role of basic psychological need satisfaction in the association between childhood harshness and mental health. Structural equation modeling was conducted using Mplus 8.3 to perform path analysis. Parameters were estimated using the maximum likelihood method, and the significance of the indirect effects was tested using a bootstrap procedure with 5000 resamples. The resulting model is presented in Figure 1. The model demonstrated a good fit to the data (RMSEA = 0.055, 90% CI [0.046, 0.064]; SRMR = 0.044; CFI = 0.982; TLI = 0.974), indicating that the proposed structural model adequately fits the sample data.

As shown in Figure 1, the path analysis results indicated that childhood harshness significantly and negatively predicted basic psychological need satisfaction (β = −0.32, *p* < 0.001), whereas basic psychological need satisfaction significantly and positively predicted mental health (β = 0.60, *p* < 0.001). After including basic psychological need satisfaction as a mediator, the direct effect of childhood harshness on mental health remained significant (β = −0.09, *p* < 0.001), indicating that basic psychological need satisfaction partially mediated the association between childhood harshness and mental health. Bootstrap analyses further indicated that the indirect effect of childhood harshness on mental health through basic psychological need satisfaction was significant (95% CI = [−0.229, −0.154]). As the confidence interval did not include zero, the mediating effect was supported. The detailed results are reported in Table 4.

## 3. Study 2

In Study 1, cross-sectional analyses indicated that childhood harshness predicted prosocial behavior, with basic psychological need satisfaction serving as a mediator. Nevertheless, due to the cross-sectional design, causal and temporal inferences could not be established. Therefore, to explicitly examine the temporal ordering of these associations, we employed a three-wave longitudinal design to investigate the prospective effects of childhood harshness on mental health and the mediating role of basic psychological need satisfaction over time.

### 3.1. Method

#### 3.1.1. Participants and Procedures

The present data were collected as part of a study conducted between October 2024 and 2025, designed to extend our prior findings in an independent sample and to further examine the temporal effects underlying the association between childhood harshness and adolescent mental health. In addition, faced with a crisis of reproducibility crisis in psychology research (Patil et al., 2016), we used a different questionnaire to measure participants’ basic psychological need satisfaction in Study 2. A total of 1207 Grade 7 students were recruited using convenience sampling from a public middle school in Dalian, Liaoning Province, China. After obtaining informed consent from both the participants and their guardians, a survey was conducted over 8 months. Data were collected in three waves, Time 1 (T1) on 9 September 2024, Time 2 (T2) on 10 January 2025, and Time 3 (T3) on 11 June 2025, with 4- to 5-month intervals between waves. These intervals align with the typical academic term length in China and allow for the observation of changes over a full semester. The data from the three waves were matched and screened for quality. Invalid questionnaires were excluded based on factors such as illness, transfer, or voluntary withdrawal. The final sample consisted of 918 valid participants, including 415 males and 503 females. The descriptive data for age are M = 12.62 years, SD = 0.61. The surveys were administered electronically via the school’s psychological health platform, guided by trained psychological teachers. The study followed strict confidentiality protocols and received approval from the Ethics Committee of Northeast Normal University (approval number: 2024014).

#### 3.1.2. Measures

##### Childhood Harshness

Childhood harshness was evaluated by the 11 items of the Chinese childhood harshness scale (Lin et al., 2024), the same as Study 1. The Cronbach’s alpha of this scale was 0.83 at T1.

##### Basic Psychological Need Satisfaction

The Basic Psychological Need Satisfaction and Frustration Scale (BPNSFS; Chen et al., 2015), using the Chinese version translated and validated by Liu et al. (2025), consists of six subscales assessing autonomy, relatedness, and competence in terms of both satisfaction and frustration, with four items in each subscale. Participants can rate the items on a 5-point Likert type scale (1 = ‘not true at all’ to 5 = ‘completely true’). For the present study, only the satisfaction subscales were used. Higher scores on the autonomy, relatedness and competence satisfaction subscales indicate greater need satisfaction. The scale has demonstrated good reliability and validity among adolescents (Turan, 2025). At T2, the Cronbach’s alpha for the overall scale was 0.95; the reliabilities of the relatedness, autonomy, and competence satisfaction subscales were α = 0.87, 0.86, and 0.90, respectively.

##### Mental Health

Mental health was evaluated by the 14 items of the mental health (Guo et al., 2015) same as Study 1. At T3, the Cronbach’s alpha for the overall scale was 0.95; the reliabilities of the emotional, social, and psychological well-being subscales were ω = 0.92, 0.87, and 0.92, respectively.

#### 3.1.3. Data Analysis

Similar to the analytic procedure in Study 1, data in Study 2 were analyzed using SPSS 26.0 and Mplus 8.3. First, descriptive statistics and Pearson correlation analyses were conducted in SPSS 26.0 to examine the associations among childhood harshness at Time 1 (T1), basic psychological need satisfaction at Time 2 (T2), and mental health at Time 3 (T3). Independent-samples *t* tests were then performed to assess gender differences. Subsequently, multiple regression analyses were conducted to examine whether childhood harshness (T1) and basic psychological need satisfaction (T2) prospectively predicted mental health (T3), while controlling for demographic variables (gender and age). Finally, structural equation modeling was conducted in Mplus 8.3 to test the longitudinal mediating role of basic psychological need satisfaction (T2) in the association between childhood harshness (T1) and mental health (T3). The significance of the indirect effect was evaluated using a bias-corrected bootstrap method.

### 3.2. Results

#### 3.2.1. Descriptive Statistics

Table 5 presents the correlational analysis among all assessed variables. The primary research variables in this study are significantly correlated.

Independent-samples *t* tests were conducted to examine gender differences across the study variables (see Table 6). The results indicated significant gender differences in childhood harshness and emotional well-being, with males reporting higher levels than females.

#### 3.2.2. Regression Analysis

Multiple linear regression analyses (enter method) were conducted with emotional, social, and psychological well-being at T3 as dependent variables, and childhood harshness at T1 together with autonomy, competence, and relatedness satisfaction at T2 as predictors. As shown in Table 7, childhood harshness negatively predicted emotional (B = −0.05, *p* < 0.05), social (B = −0.09, *p* < 0.01), and psychological well-being (B = −0.07, *p* < 0.01). In contrast, autonomy, competence, and relatedness satisfaction positively predicted all three dimensions of mental health, with competence satisfaction demonstrating the strongest unique predictive effect across models (partial r ranging from 0.53 to 0.68).

#### 3.2.3. Mediation Analysis

Extending the cross-sectional findings of Study 1, we further tested a temporal mediation model to examine whether basic psychological need satisfaction at T2 mediated the longitudinal association between childhood harshness at T1 and mental health at T3. Structural equation modeling was conducted in Mplus 8.3, with parameters estimated using the maximum likelihood method. Indirect effects were evaluated using bias-corrected bootstrap procedures with 5000 resamples. The model demonstrated a good fit to the data (RMSEA = 0.049, 90% CI [0.037, 0.061]; SRMR = 0.032; CFI = 0.989; TLI = 0.984), indicating that the proposed longitudinal structural model adequately captured the temporal relationships among the variables.

**Figure 2:**
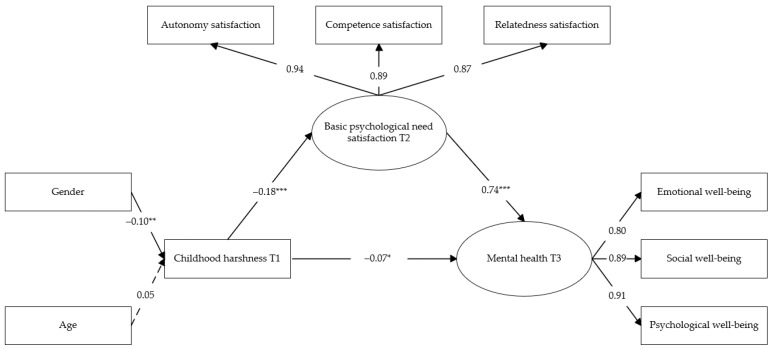
A mediation model of childhood harshness, basic psychological need satisfaction, and mental health in Study 2. Note. * *p* < 0.05, ** *p* < 0.01, *** *p* < 0.001.

As shown in Figure 2, the path analysis indicated that childhood harshness at T1 significantly and negatively predicted basic psychological need satisfaction at T2 (β = −0.18, *p* < 0.001), which in turn significantly and positively predicted mental health at T3 (β = 0.74, *p* < 0.001). After including basic psychological need satisfaction as a mediator, the direct effect of childhood harshness at T1 on mental health at T3 remained significant (β = −0.07, *p* < 0.05), suggesting a partial mediation effect. Bootstrap analyses further demonstrated that the indirect effect of childhood harshness at T1 on mental health at T3 through basic psychological need satisfaction at T2 was significant (95% CI = [−0.186, −0.076]). Because the confidence interval did not include zero, the mediating effect was supported. Detailed results are presented in Table 8.

## 4. Discussion

The present research aimed to examine the relationship between childhood harshness and mental health among adolescents, as well as to explore its underlying mechanisms. Two retrospective studies were conducted. As predicted, both Study 1 and Study 2 consistently found that higher childhood harshness was associated with poorer mental health, even after controlling for age and gender. Additionally, both studies demonstrated that the effect of childhood harshness on adolescent mental health was mediated by basic psychological need satisfaction.

### 4.1. The Relation Between Childhood Harshness and Adolescents’ Mental Health

Although a substantial body of literature has documented associations between broader childhood adversity and adolescent mental health (Bakker et al., 2012), the specific relationship between childhood harshness and distinct dimensions of positive mental health has received comparatively limited empirical attention. The present findings indicate that childhood harshness was significantly negatively associated with emotional well-being, psychological well-being, and social well-being. Adolescents exposed to higher levels of childhood harshness may develop heightened needs for care and external support and may be more inclined to adopt a fast life history strategy (Chang et al., 2019; Grossmann & Varnum, 2011). Such strategies, which prioritize immediate survival over long-term investment, may be adaptive in ecologically harsh environments. However, in socially structured contexts that emphasize emotional stability, future planning, and sustained relational engagement, these tendencies may undermine long-term functioning (Del Giudice & Ellis, 2016). This mismatch may help explain the observed reductions across multiple domains of well-being. While much of the prior research has relied on cross-sectional studies (M. Yang et al., 2024; Zhang et al., 2024), the present study extends and integrates prior work through a combined cross-sectional and longitudinal design, with findings validated across different adolescent samples. This approach enhances the robustness of our conclusions and provides stronger support for the proposed associations. Our results further indicate that higher levels of childhood harshness are associated with poorer mental health during adolescence, with potential implications for both short-term and longer-term well-being. Moreover, even after controlling for demographic variables, childhood harshness remained a significant negative predictor of emotional well-being, psychological well-being, and social well-being, underscoring the robustness and persistence of these associations over time.

Our findings contribute new insights into Life History Theory by demonstrating that individuals who experience childhood harshness tend to adopt faster life history strategies, which may hinder the development of essential psychological resources, such as emotional regulation and social support. These resources are critical for maintaining healthy mental functioning and adaptive social behavior, especially in adolescence (Hurst & Kavanagh, 2017; Zhang et al., 2023). This process of adaptive maladaptation further complicates the mental health outcomes in adolescents from harsh backgrounds, highlighting the need for interventions that address the specific challenges posed by fast life history strategies in these environments.

### 4.2. Childhood Harshness, Basic Psychological Need Satisfaction and Adolescents’ Mental Health

As hypothesized, basic psychological need satisfaction mediated the relationship between childhood harshness and adolescents’ mental health in both Study 1 and Study 2. Furthermore, this mediation effect remained significant even after controlling for gender and age across both cross-sectional and longitudinal samples. Adolescents with higher levels of childhood harshness were found to exhibit lower levels of basic psychological need satisfaction, which, in turn, negatively impacted their mental health.

Consistent with previous research, we found that childhood harshness negatively predicted the satisfaction of basic psychological needs. Studies have shown that positive life attitudes and adaptive coping abilities (such as relatedness, social participation, and effective coping strategies) are not necessarily related to the severity of trauma but are more closely tied to one’s immediate environment (Lera & Abualkibash, 2022). Childhood harshness, characterized by deprivation and scarcity, represents an environment where individuals prioritize survival over psychological well-being, making it challenging to fulfill basic psychological needs. Furthermore, the negative predictive relationship between childhood harshness and basic psychological need satisfaction supports the Basic Psychological Need Satisfaction Theory (Ryan & Deci, 2000), indicating that harsh environments hinder the fulfillment of individuals’ basic psychological. Childhood harshness can make individuals more prone to risk-taking behaviors, increased aggression, early sexual behavior, and decreased empathy (Del Giudice, 2014). While these behaviors may be adaptive from an evolutionary perspective, they may not be considered ideal from a mental health standpoint and are often viewed as symptoms of psychopathology. This negative interaction pattern may increase the likelihood of rejection by family and society, leading to lower levels of relatedness need satisfaction in abused adolescents (Gu et al., 2023).

This study found that satisfaction of all three basic psychological needs was significantly associated with adolescents’ mental health, and lower levels of need satisfaction further predicted declines in emotional, psychological, and social well-being. Previous research has similarly demonstrated that autonomy, competence, and relatedness satisfaction are essential for sustaining long-term well-being (Lataster et al., 2022). When these needs are adequately fulfilled, individuals are more likely to display adaptive behavioral patterns and experience greater vitality and psychological flourishing, which provide important resources for coping with distress (Jovanović et al., 2024). Taken together, our findings highlight childhood harshness as a significant negative predictor of adolescents’ basic psychological need satisfaction and reaffirm the protective role of need satisfaction in promoting multiple dimensions of adolescent mental health.

More importantly, our study is the first to uncover the mediating role of basic psychological need satisfaction in the relationship between childhood harshness and adolescent mental health. According to Life History Theory, individuals who experience early life adversity, such as childhood harshness, may adopt faster life history strategies, which prioritize short-term survival and resource acquisition over long-term well-being (Belsky et al., 1991). This accelerated strategy can hinder the development of secure attachment relationships, leading to unmet basic psychological needs, such as competence, autonomy, and relatedness (Deci & Ryan, 2000). When these needs remain unmet, adolescents may develop maladaptive coping mechanisms, such as emotional dysregulation or externalizing behaviors, which in turn negatively impact their mental health. Therefore, our results emphasize the critical importance of addressing unmet psychological needs in intervention programs aimed at improving adolescent mental health, particularly for those who have experienced childhood adversity.

### 4.3. Limitations and Future Directions

Several limitations of our study should be noted. First, both Study 1 and Study 2 involved only Chinese participants. Basic psychological needs may vary across cultures, which could affect the generalizability of our findings (Iyengar & DeVoe, 2003). Therefore, future research could explore the universality of these findings in different cultural contexts. Second, the participants in this study were students from general schools who were asked to report their childhood experiences of harshness. The results may not be applicable to vulnerable student groups who face a higher risk of childhood adversity. Future research should include more diverse populations to enhance the external validity of the findings. Third, this study employed a retrospective design. Although we combined cross-sectional and longitudinal methods to minimize recall bias, the data were entirely self-reported. Future research could incorporate reports from multiple sources, such as parents, teachers, and self-reports, to validate the unique impact of childhood harshness on adolescent mental health and increase the objectivity of the findings. Additionally, further longitudinal studies with more measurement points are needed to assess the relationships between the study variables over time.

### 4.4. Implications

Despite these limitations, the current study has significant implications both theoretically and practically.

From a theoretical perspective, our study, using both cross-sectional and longitudinal methods, found that childhood harshness negatively predicts adolescent mental health outcomes. We also explored the mediating role of basic psychological need satisfaction. The results provide empirical support for life history theory and self-determination theory, offering deeper insights into the mechanisms through which early adversity affects psychological well-being in adolescence.

From a practical perspective, given the long-term impact of childhood adversity, it is crucial for caregivers to recognize the significant role of the early environment in shaping adolescent development. Childhood harshness, especially during critical periods of physical and psychological growth, can have lasting effects on adolescents’ mental health and overall well-being. Therefore, caregivers should be encouraged to provide a supportive, nurturing environment that fosters emotional security and resilience in children. Since basic psychological need satisfaction mediates this relationship, schools can support adolescents with high adversity by offering interventions that enhance their sense of competence, autonomy, and relatedness. For example, programs promoting emotional regulation and positive peer interactions can help fulfill these needs and improve mental health outcomes.

## 5. Conclusions

In summary, using a sample of Chinese participants, the present study systematically examined the association between childhood harshness and adolescent mental health across both short-term and longer-term time frames. The regression analyses demonstrated that childhood harshness was consistently associated with lower levels of mental health at multiple levels, and these associations were evident in both short-term and longitudinal contexts. At the same time, all dimensions of basic psychological need satisfaction were positively associated with higher levels of mental health, underscoring the central role of autonomy, competence, and relatedness in adolescents’ psychological functioning. Further structural equation modeling conducted in Mplus revealed that basic psychological need satisfaction served as a significant mediator in the relationship between childhood harshness and adolescent mental health. These findings suggest that childhood harshness may undermine adolescents’ mental health partly by frustrating their fundamental psychological needs. By examining the impact of childhood harshness and its indirect pathway through basic psychological need satisfaction, this study offers additional evidence regarding how early harsh environments may affect adolescent mental health.

Overall, the findings deepen our understanding of the long-term implications of childhood harshness and highlight the importance of interventions that target the satisfaction of basic psychological needs as a potentially effective pathway to promote adolescent mental health.

## Figures and Tables

**Table 1:** Descriptive statistics for the variables in Study 1.

	M	SD	1	2	3	4	5	6	7
1. Childhood harshness	2.98	1.17	1						
2. Autonomy satisfaction	5.39	1.44	−0.27 ***	1					
3. Competence satisfaction	5.18	1.36	−0.27 ***	0.74 ***	1				
4. Relatedness satisfaction	5.98	1.18	−0.28 ***	0.74 ***	0.67 ***	1			
5. Emotional well-being	4.73	1.11	−0.23 ***	0.49 ***	0.50 ***	0.47 ***	1		
6. Social well-being	4.55	1.18	−0.23 ***	0.44 ***	0.48 ***	0.45 ***	0.71 ***	1	
7. Psychological well-being	4.79	1.14	−0.26 ***	0.45 ***	0.51 ***	0.45 ***	0.71 ***	0.80 ***	1

Note. *** *p* < 0.001.

**Table 2:** Gender differences across variables in Study 1.

Variables	Male (*n* = 759)	Female (*n* = 731)	t	*p*
Childhood harshness	3.10 ± 1.19	2.86 ± 1.13	3.84	0.000
Autonomy satisfaction	5.39 ± 1.49	5.39 ± 1.49	0.215	0.830
Competence satisfaction	5.18 ± 1.36	5.18 ± 1.37	0.094	0.925
Relatedness satisfaction	6.04 ± 1.09	5.91 ± 1.27	2.129	0.033
Emotional well-being	4.79 ± 1.07	4.67 ± 1.14	2.132	0.033
Social well-being	4.60 ± 1.13	4.50 ± 1.22	1.632	0.103
Psychological well-being	4.82 ± 1.11	4.76 ± 1.18	0.980	0.327

**Table 3:** Regression Analysis of Childhood Harshness and Basic Psychological Need Satisfaction on Mental Health in Study 1.

Dependent Variable	Predictor	Partial r	B	*p*	*R* ^2^
Emotional well-being	Childhood harshness	−0.24	−0.08	0.000	0.30
	Autonomy satisfaction	0.48	0.13	0.000	
	Competence satisfaction	0.49	0.2	0.000	
	Relatedness satisfaction	0.46	0.14	0.000	
Social well-being	Childhood harshness	−0.24	−0.09	0.000	0.27
	Autonomy satisfaction	0.44	0.07	0.020	
	Competence satisfaction	0.48	0.24	0.000	
	Relatedness satisfaction	0.44	0.17	0.000	
Psychological well-being	Childhood harshness	−0.27	−0.12	0.000	0.29
	Autonomy satisfaction	0.45	0.06	0.061	
	Competence satisfaction	0.50	0.27	0.000	
	Relatedness satisfaction	0.44	0.14	0.000	
Step 1: Method = Enter					

**Table 4:** Bootstrap analysis of the mediated effect test for childhood harshness and mental health in Study 1.

Effect	Effect Value	Boot SE	Boot CI Lower	Boot CI Upper	Effect Rate, %
Direct effect	−0.091	0.026	−0.143	−0.040	32.38%
Indirect effect	−0.190	0.019	−0.229	−0.154	67.62%
Total effect	−0.281	0.029	−0.338	−0.223	-

**Table 5:** Descriptive statistics for the variables in Study 2.

	M	SD	1	2	3	4	5	6	7
1. Childhood harshness T1	2.99	1.05	1						
2. Autonomy satisfaction T2	4.04	0.78	−0.16 ***	1					
3. Relatedness satisfaction T2	4.14	0.75	−0.16 ***	0.83 ***	1				
4. Competence satisfaction T2	3.98	0.84	−0.16 ***	0.84 ***	0.76 ***	1			
5. Emotional well-being T3	4.97	1.02	−0.15 ***	0.55 ***	0.51 ***	0.54 ***	1		
6. Social well-being T3	4.58	1.23	−0.18 ***	0.59 ***	0.55 ***	0.60 ***	0.73 ***	1	
7. Psychological well-being T3	4.90	1.09	−0.18 ***	0.65 ***	0.60 ***	0.68 ***	0.72 ***	0.81 ***	1

Note. *** *p* < 0.001.

**Table 6:** Gender differences across variables in Study 2.

Variables	Male (*n* = 759)	Female (*n* = 731)	t	*p*
Childhood harshness T1	3.11 ± 1.07	2.90 ± 1.02	3.070	0.002
Autonomy satisfaction T2	4.08 ± 0.74	4.00 ± 0.81	1.398	0.162
Relatedness satisfaction T2	4.16 ± 0.74	4.12 ± 0.76	0.641	0.539
Competence satisfaction T2	4.03 ± 0.79	3.95 ± 0.87	1.465	0.143
Emotional well-being T3	5.05 ± 0.97	4.90 ± 1.06	2.182	0.029
Social well-being T3	4.66 ± 1.18	4.51 ± 1.27	1.799	0.072
Psychological well-being T3	4.96 ± 1.06	4.85 ± 1.12	1.490	0.137

**Table 7:** Regression Analysis of Childhood Harshness and Basic Psychological Need Satisfaction on Mental Health in Study 2.

Dependent Variable	Predictor	Partial r	B	*p*	*R* ^2^
Emotional well-being T3	Childhood harshness T1	−0.15	−0.05	0.049	0.33
	Autonomy satisfaction T2	0.55	0.36	0.000	
	Competence satisfaction T2	0.53	0.26	0.000	
	Relatedness satisfaction T2	0.50	0.14	0.040	
Social well-being T3	Childhood harshness T1	−0.18	−0.09	0.004	0.40
	Autonomy satisfaction T2	0.59	0.32	0.000	
	Competence satisfaction T2	0.60	0.48	0.000	
	Relatedness satisfaction T2	0.55	0.2	0.010	
Psychological well-being T3	Childhood harshness T1	−0.19	−0.07	0.005	0.49
	Autonomy satisfaction T2	0.65	0.27	0.000	
	Competence satisfaction T2	0.68	0.56	0.000	
	Relatedness satisfaction T2	0.60	0.16	0.013	
Step 1: Method = Enter					

**Table 8:** Bootstrap analysis of the mediated effect test for childhood harshness and mental health in Study 2.

Effect	Effect Value	Boot SE	Boot CI Lower	Boot CI Upper	Effect Rate, %
Direct effect	−0.065	0.029	−0.124	−0.007	33.16%
Indirect effect	−0.131	0.028	−0.186	−0.076	66.84%
Total effect	−0.196	0.037	−0.269	−0.125	-

## Data Availability

The data that support the findings of this study are available from the corresponding author upon reasonable request.

## Abbreviation

The following abbreviation is used in this manuscript:

BPNS	Basic Psychological Need Satisfaction
